# Impact of social violence and childhood adversities on pregnancy outcomes: a longitudinal study in Tunisia

**DOI:** 10.7189/jogh.09.020435

**Published:** 2019-12

**Authors:** Arwa Ben Salah, Andrine Lemieux, Imen Mlouki, Ines Amor, Ines Bouanene, Kamel Ben Salem, Mustafa al’Absi, Sana El Mhamdi

**Affiliations:** 1 Department of Community Medicine, Faculty of Medicine, University of Monastir, Monastir, Tunisia; 2 Research laboratory “Epidemiology Applied to Maternal and Child Health”, Monastir, Tunisia; 3 Department of Family Medicine and Biobehavioral Health, University of Minnesota Medical School, Duluth, Minnesota, USA; 4 Department of Preventive and Community Medicine, University Hospital Tahar Sfar, Mahdia Tunisia

## Abstract

**Background:**

Accumulating research suggests that exposure to intra-familial adversities are significant risk factors for adverse pregnancy outcomes. However, the relationship between social violence (peer violence, witnessing community violence and exposure to collective violence) and pregnancy outcomes has not been extensively investigated. Our study aims to examine the association between social Adverse Childhood Experiences (ACEs) and pregnancy outcomes and to explore the role of depression during pregnancy as a mediator of this association.

**Methods:**

We performed a prospective follow-up study of pregnant women in five Primary Health care Centers (PHC) in the region of Monastir (Tunisia) from September 2015 to August 2016. Enrolled women were followed during the second trimester, third trimester of pregnancy and during the postnatal period. Exposure to violence was assessed retrospectively using the validated Arabic version of the World Health Organization (WHO) ACE questionnaire. The Self Reporting Questionnaire 20-Item (SRQ-20) was used as a screening tool for depression during pregnancy.

**Results:**

We recruited and followed a total of 593 women during the study period. Witnessing community violence was the most frequently reported social ACE among pregnant women (237; 40%), followed by peer violence (233; 39.3%). After adjustment for high risk pregnancies, environmental tobacco smoke, and intra-familial ACEs, the risk of premature birth was significantly associated with exposure to collective violence (*P* < 0.001) and witnessing community violence (*P* < 0.05). The risk of low birth weight was significantly associated with witnessing community violence (*P* < 0.001). In the mediation analysis, depression mediated significant proportions of the relationship between the cumulative number of ACEs and pregnancy outcomes.

**Conclusions:**

Social ACEs may have a long-term effect on maternal reproductive health, as manifested by offspring that were of reduced birth weight and shorter gestational age. A public health framework based on the collaboration between pediatric, psychiatric obstetrical health professionals, education professionals and policy makers could be applied to ensure primary prevention of childhood adversities and pay attention to expected mothers with history of exposure to such adversities.

The early years of a child’s life are fundamentally important in shaping his/her future health and well-being. They have an extended influence on academic achievement, on the strength of relationships with parents, and on ties to the community [1]. Individuals' childhood experiences play a key role in affecting health and social outcomes through the life course. Growing evidence suggests that adverse childhood experiences (ACEs) that occur prior to the age of 18 years can lead to significant physical and mental disorders, early mortality, and increased long-term chronic diseases (eg, chronic obstructive pulmonary disorder, cardiovascular diseases, and mental disorder) [2-4]. Increasing exposure to such adversities has also been associated with escalating risk of health harming behaviors, such as illicit drug use, alcohol abuse, violence and suicidal behaviors [5,6].

Recent studies have even found a significant association between ACEs and pregnancy outcomes [7-9]. The majority of these studies have focused on intra-familial childhood adversities in the form of emotional and physical neglect, emotional and physical abuse, household dysfunction, and child sexual abuse (CSA) [10,11]. Studies have also reported a dose-response relationship between the number of reported ACEs and the risk of adverse outcomes. Retrospective, as well as prospective, studies have confirmed an association between CSA experiences and the risk for premature birth [8,9,12], as well as infant admission to neonatal intensive care unit and maternal and infant health complications after childbirth [9,13]. Suicidal ideation and depressive symptoms during pregnancy have also been associated with the number of adversities experienced during childhood [8,14]. In addition, lifelong abusive experiences can affect women throughout the gravidness cycle. It is currently known that many physical, hormonal, and neurochemical changes occur during the perinatal period making the mother more vulnerable to depression [13,15]. Many research papers have also reported an association of maternal history of childhood abuse, especially physical abuse [16], sexual abuse [10] and postpartum depression [17-20].

In the same context, the health and well-being of children and adolescents require stability, security, and positive connection to the community. These basic needs are jeopardized due to the deterioration of social connections present in Middle East countries, specifically the outbreaks of social violence. Currently, there is an increasing awareness of the public health impact of social violence [21,22]. It is among the top twenty causes of worldwide loss of disability-adjusted living years (DALY), and projected to increase in importance by 2030 according to the World Health Organization (WHO) [23].

Social violence refers to any kind of violence committed by individuals outside of the family unit within the community that has a serious social impact. These violent acts take diverse forms, including armed conflicts, gang violence, bullying or peer victimization, terrorism, deportation, and social segregation. Experiencing social violence can be direct such as being the victim of violent acts or indirect such as witnessing or hearing about violence including others. Researchers have produced a vast body of work exploring the potential consequences of social violence on mental health. A dose-response relationship has been observed between increasing frequency of victimization by peers in adolescence and the risk of developing depression, anxiety and suicidality [24-27]. In addition, living in violent communities increases the likelihood of developing internalizing mental disorders such as anxiety, depression, and posttraumatic stress disorder (PTSD) [28,29]. The impact of social violence-related depression, anxiety, and PTSD on pregnancy outcomes has been studied relative to peer and community violence in western cultures [30], but rarely has it been studied in the context of exposure to collective violence such as war, social unrest, or political transition.

Despite the increasing community violence (seeing or hearing someone being beaten up, someone being stabbed or shot or someone being threatened with a knife or gun in real life) and collective violence (being beaten up soldiers, police, militia, or gangs) worldwide, most importantly in the Middle East and in Africa, the impact of social violence and childhood adversities on later pregnancy outcomes and depression during pregnancy and postpartum period has not been systematically evaluated. Since the 2011 Tunisian revolution and the resulting multi-level crises that occurred, the government, the economy, and the national security have been challenged by a serious increase in social violence. For instance, Tunisia has seen an **increase** since the revolution of armed conflicts, terrorist attacks, the spread of crimes like homicides, child kidnapping and rape, along with behavioral problems such as interpersonal hostility, illicit drug use, alcohol abuse and suicidality. Such social violence has been shown to double (peer and community violence) and triple (collective violence) the risk of addictive behaviors in Tunisian women [31]. Whether similar impact is evident in pregnancy outcomes has not been examined.

The aim of the current study was to assess the relation between social violence experienced during childhood or adolescence and subsequent adverse pregnancy outcomes. While the question at hand addresses impact of early adversity, the current situation in the country provides a context with increased societal stress that may accentuate impact of early adversity. We hypothesized that social violence would affect pregnancy outcomes after controlling for other childhood adversities and pregnancy risks. We also sought to investigate the role of depression during pregnancy as a mediator of this association.

## METHODS

### Study design

**Figure 1:**
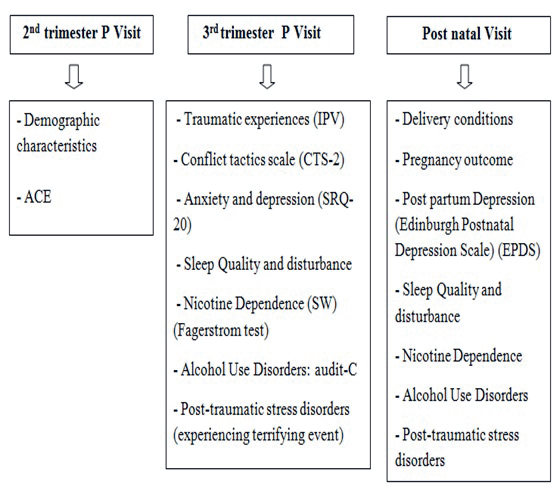
The schedule of the visits performed by the midwives.

A prospective study was conducted among pregnant women in five Primary Health care Centers (PHC) in the region of Monastir (Tunisia) from September 2015 to August 2016. Enrolled women were followed three times during the second trimester, third trimester and during the postnatal period. Data were collected by trained midwives according to a pre-established schedule (**Figure 1**).

### Inclusion/exclusion criteria

Women were eligible to participate if they were at least 18 years of age and were willing to provide written informed consent. Women were ineligible if they had a multi-fetal pregnancy, intended to terminate their pregnancy, or had a known Fetal Growth Retardation (FGR).

### Study instruments

#### Measurement of childhood adversities

Adverse childhood experiences were evaluated using the Arab version of the ACE-International Questionnaire (ACE-IQ) developed by WHO [32]. This version was validated in Saudi Arabia [33], and then adapted to the Tunisian context. It includes eight categories of ACEs divided in two sections:

- Intra-familial ACEs: including conflictual relationship to parents/caregivers; neglect; household dysfunction; physical abuse; sexual abuse.
- Social ACEs (ACEs experienced in the society): including peer violence; witnessing community violence and exposure to war/collective violence.

This Arabic ACE-IQ tool also includes questions about chronic health conditions (diabetes, hypertension, cancer…), depression, anxiety and other mental illnesses. All questions about ACEs were related to the respondents’ first 18 years of life (Appendix S1 in **Online Supplementary Document**).

#### Measurement of pregnancy outcomes

We measured gestational age at delivery (completed gestational weeks). Birth weight and Apgar score were extracted from the pregnant medical record. The risk of prematurity was defined as a gestational age <37 weeks, low birth weight (LBW) was defined as weight <2500 gr, and acute fetal distress was defined as an Apgar score at 5 minutes of less than 7 [34].

#### Measurement of demographic, obstetric and clinical characteristics

A structured questionnaire designed at the Department of Community Medicine of Monastir was used to collect socio-demographic data (age, marital status and personal history) and obstetrical characteristics. A pregnancy was considered a 'high risk pregnancy' if the mother was an extreme age (<19 years old or ≥35 years old), had diabetes or high blood pressure, or she was multiparous. Current active smoking and environmental tobacco smoke (ETS), alcohol abuse, illicit drug use and risky sexual behaviors were also assessed by self-report. Regarding exposure to ETS, nonsmoking mothers were asked if they were regularly exposed to ETS, where and by whom (husband or other family member or/and at work). The mean number of cigarettes and hours of exposure were also estimated.

Common Mental Disorders (CMD), including depression and anxiety, were evaluated using an Arab version of the 20-item Self-Reporting Questionnaire (SRQ-20). It is a screening instrument developed by the WHO for use in community and primary care settings, especially in developing countries, to identify symptoms indicative of anxiety or depression (probable diagnosis score >7) [35].

### Data collection and reporting procedures

At each assessment visit (during the second trimester, the third trimester and during the postnatal period), we collected data about depressive symptoms, tobacco and alcohol during pregnancy. We also collected data on psychiatric medication use and pregnancy complications. Information about possible exposure to adverse childhood events during the first 18 years of life was assessed by administrating the ACE-IQ during the first follow-up visit.

### Ethical approval

The study was approved by the local ethics committee of the university hospital of Monastir. Women gave their informed written consent to participate. Each woman was informed that participation was voluntary and could be withdrawn any time.

### Statistical analyses

Data collection and analysis were performed using the Statistical Package for Social Sciences (SPSS) version 21.0. The descriptive analysis involved an examination of the demographic characteristics of the study sample. Univariate and multivariate linear regression analyses were performed to estimate the relationship between gestational age, birth weight and social violence. We also performed a univariate and multivariate logistic regression analysis to assess the relationship between social violence and acute fetal distress.

Spearman correlations were used to assess the zero-order relationships among social ACEs, depression during pregnancy, and pregnancy outcomes. Mediation modeling was performed to determine the presence of a significant mediation (or indirect effect) of depression during pregnancy in the relationship between social ACEs and pregnancy outcomes using the linear regression approach advocated by Hayes [36]. Depression was considered as a potential mediating variable when the inclusion of depression into the model resulted in a partial or total diminution of the relationship between social ACEs as the independent variable and pregnancy outcomes was the dependent variable [37]. Mediation analyses were conducted using the PROCESS macro developed by Andrew F. Hayes [36]. Results were also adjusted to ETS, intra-familial ACEs and high-risk pregnancies. Verification of the indirect effect was assessed using the Sobel test [38].

**Figure 2:**
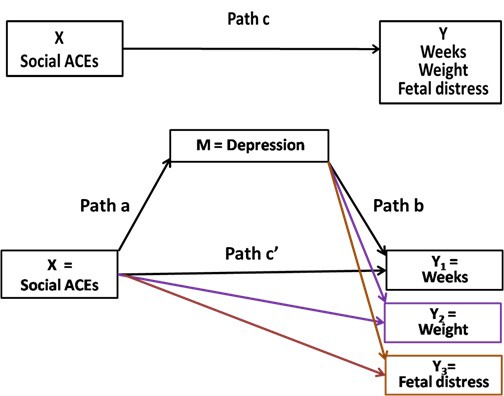
Diagram of the theoretical relationships between social ACEs (cumulative number), depression during pregnancy, and pregnancy outcomes.

To apply the mediation model, some criteria must be satisfied (**Figure 2**). First, the relationship between the independent variable (social ACEs) and the dependent variable (each pregnancy outcome) must be significant **(**pathway a**)**; second, the variable of mediation (depression) must be significantly associated with each pregnancy outcome and with the social ACEs **(**pathway b**)**; and finally, the relationship between the social ACEs and depression must be significant **(**pathway c**)**. Significant mediation occurs when pathway c is reduced significantly (partial mediation) or no longer significant (full mediation) by the inclusion of the mediator into the assessment of pathway c (pathway c’). A *P*-value less than .05 was considered as statistically significant.

## RESULTS

A total of 593 pregnant women were enrolled during the study period. **Table 1** describes the demographic characteristics and neonatal features of the study population according to the occurrence of social ACEs. The mean age was 28.9 ± 5.2 for women reporting at least one social ACE and 29.5 ± 5.3 for women without social ACEs history (*P* = 0.09). No significant differences were found in the two groups of women regarding the history of abortion or risky health conditions during pregnancy. ETS was higher among women exposed to at least one social ACE (*P* = 0.004) but none of our women reported personal use of tobacco. The SRQ-20 depression score was also significantly higher among women who reported at least one social ACE (*P* < 0.0001). The distribution of pregnancy outcomes showed that prematurity and fetal distress were more prevalent among women with social ACEs history (*P* < 0.0001). The mean birth weight was 3110 ± 614gr for women with a history social ACEs and 3275 ± 473gr for the women without such a history (*P* = 0.001) (**Table 1**).

**Table 1:** Demographic characteristics and neonatal features of the study population. Adverse Childhood Experiences (ACEs study) among pregnant women in Tunisia; 2016

Demographic characteristics and neonatal features	No social ACEs (n = 228)	≥1 social ACE (n = 365)	*P*-value
Age (years)	29.5 ± 5.3	28.9 ± 5.2	0.09
Unmarried women (n; %)	0	2 (.6%)	0.52
High risk pregnancy (n; %)	55 (24.1)	97 (26.6)	0.56
History of abortions (n; %)	72 (32.1)	106 (29.2)	0.46
Active tobacco smoke (n; %)	0	0	–
Environmental tobacco smoke (n; %)	106 (46.5)	215 (58.9)	0.004
Alcohol and substance use (n; %)	0	0	–
Score of depression (means ± SD)	4.6 ± 3.9	7.9 ± 4.3	<0.0001
Completed gestational weeks (means ± SD)	38.9 ± 1.9	37.3 ± 1.4	<0.0001
Birth weight (means ± SD)	3275 ± 473	3110 ± 614	0.001
Fetal distress	9 (3.9)	48 (13.2)	<0.0001

ACE – adverse childhood experience, SD – standard deviation

Among this sample of pregnant women, 228 (38.4%) reported no history of social ACEs and 219 (36.9%) reported exposure to one social adversity during the first 18 years of their life. The exposure to the three types of social ACEs was reported by 19 (3.2%) women (**Table 2**).

**Table 2:** The total number of ACE categories reported; Adverse Childhood Experiences (ACEs) study among pregnant women in Tunisia 2016

Number of social ACEs	Number	%
No social ACEs	228	38.4
One social ACE	219	36.9
Two social ACEs	127	21.4
Three social ACEs	19	3.2
**Total**	593	100

ACE – adverse childhood experience

Childhood adversities were common (**Table 3**). In fact, 88.9% of women reported a history of exposure to at least one ACE. Among the recruited women, 277 (46.7%) reported three or more ACEs. Among intra-familial ACEs, household dysfunction was the most frequent ACE category with 346 (58.3%) women followed by physical abuse and emotional abuse at 40% and 32.2% respectively (**Table 3**). The distribution of social ACEs is also included in **Table 3**. Witnessing community violence was the most reported social ACE among pregnant women (237; 40%) followed by peer violence (233; 39.3%). Exposure to war/collective violence was the least frequent social ACE (60; 10.1%).

**Table 3:** Number of women reporting each Adverse Childhood Experience (ACE) category. ACEs study among pregnant women in Tunisia, 2016

Total ACEs	Number	%	
No ACEs	66	11.1	
One ACE	121	20.4	
Two ACEs	129	21.8	
≥Three ACEs	277	46.7	
**ACE by type:**			
**Intra-familial ACEs**			
Emotional neglect	86	14.5	
Physical neglect	65	11	
Emotional abuse	191	32.2	
Sexual abuse	51	8.6	
Physical abuse	237	40	
Household dysfunction	346	58.3	
**Social ACEs:**			
Peer violence	233		39.3
Witnessing community violence	237		40
Exposure to war/collective violence	60		10.1

ACE – adverse childhood experience

### Regression analysis of social ACEs and pregnancy outcomes

**Table 4** depicts the analysis of the relationship between the three categories of social ACEs and the adverse pregnancy outcomes. In the unadjusted model, exposure to peer and collective violence were significantly associated with the occurrence of a premature birth. In fact, exposure to peer violence was significantly associated with fewer number of gestational weeks (β = -0.39; 95% CI = -0.69, -0.09, *P* < 0.01) and collective violence also decreased the number of gestational weeks (β = -1.42; 95% CI = -1.9, -0.94, *P* < 0.001). There was no such relationship with exposure to community violence. After adjustment for high-risk pregnancies, environmental tobacco smoke, and intra-familial ACEs, the risk of premature birth remained significant for exposure to collective violence (β = -1.15; 95% CI = -1.69, -0.62, *P* < 0.001) and become significant for witnessing community violence (β = 0.42; 95% CI = 0.07, 0.77, *P* < 0.05). Exposure to peer violence, however, was no longer significant suggesting that the previous relationship was due to one or more of these adjustors.

**Table 4:** Unadjusted and adjusted relationships between social ACEs and pregnancy outcomes (N = 593)

Social ACEs	Gestational age (weeks) unadjusted β (95% CI)		Adjusted β (95% CI)*		Birth weight unadjusted β (95% CI)		Adjusted β (95% CI)*		Fetal distress unadjusted OR (95% CI)		Adjusted OR (95% CI)†
**Peer:**											
Yes	-0.39 (-0.69, -0.09)§	-0.11 (-0.46, 0.24)		40.6 (-85.1, 166.3)		-69.4 (-176.9, 38)		1.97 (1.13, 3.41)‡		1.03 (0.51, 2.05)	
No	Referent	Referent		Referent		Referent		Referent		Referent	
**Community:**											
Yes	0.10 (-1.95, 0.41)	0.42 (0.07, 0.77)‡		-103 (-210, -4.7)‡		-85.5 (-194.3, 23.3)		2.24 (1.28, 3.89)§		2.14 (1.07, 4.29)‡	
No	Referent	Referent		Referent		Referent		Referent		Referent	
**Collective:**											
Yes	-1.42 (-1.9, -0.94)‖		-1.15 (-1.69, -0.62)‖		-482.4 (-653.2, -311.5)‖		-456.1 (-629.5, -282.7)‖		6.04 (3.19, 11.4)‖		2.50 (1.20, 5.60)‡
No	Referent		Referent		Referent		Referent		Referent		Referent

ACE – adverse childhood experience, CI – confidence interval, OR – odds ratio

*Adjusted to high risk pregnancies, environmental tobacco smoke, and intra-familial ACEs.

†Adjusted to high risk pregnancies, environmental tobacco smoke, premature birth and intra-familial ACEs.

‡*P* < 0.05.

§*P* < 0.01.

‖*P* < 0.001.

The risk of low birth weight increased significantly among women exposed to community (β = -103; 95% CI = -210, -4.7, *P* < 0.05) and collective violence (β = -482.4; 95% CI = -653.2, -311.5, *P* < 0.001) during their childhood (unadjusted analysis). In the adjusted model, however, only exposure to collective violence remained significant. In this adjusted model, exposure to collective violence significantly reduced the birth weight by 456.1 g (95% CI = -629.5, -282.7; *P* < 0.001).

Results of the univariate logistic regression analysis showed that all categories of social ACEs significantly increased the odds of fetal distress (OR range 1.97-6.04). After adjustment to high risk pregnancies, environmental tobacco smoke, premature birth, and intra-familial ACEs, two social ACEs remain significant. In fact, witnessing community violence increased the odds of fetal distress by 2.14-fold (95% CI = 1.07, 4.29; *P* < 0.05). Exposure to collective violence increased the odds of fetal distress by 2.50-fold (95% CI = 1.20, 5.60; *P* < 0.05) (**Table 4**).

### Mediation analysis

All variables included in the mediation analysis were significantly correlated (**Table 5**). In fact, social ACEs and depression were independently associated with the three selected pregnancy outcomes (correlation range = 0.10-0.20, *P* < 0.01), and social ACEs were significantly associated with depression (*P* < 0.001).

**Table 5:** Zero-order relationships between study variables. ACE study among pregnant women in Tunisia, 2016

	(1)	(2)	(3)	(4)	(5)
(1) cumulative Social ACEs	–	0.36†	-0.15†	-0.13†	0.10†
(2) Depression		–	-0.20†	-0.16†	*-0.12*
(3) Gestational age			–	–	–
(4) Birth weight				–	–
(5) Fetal distress					–

ACE – adverse childhood experience

*P < 0.01.

†*P* < 0.001.

The results of the mediation model are displayed in **Table 6**. After adjustment for covariates (ETS, high risk pregnancies, and intra-familial ACEs) and accounting for the indirect effect of depression during pregnancy, statistically significant partial mediation effects were observed for the cumulative number of social ACEs as the exposure variable and premature birth as the outcome variable (*P* = 0.0033; % mediated = 93%). No statistically significant indirect effects were found for social ACEs in their relationship with low birth weight (*P* = 0.054; % mediated = 28.8%) and with fetal distress as a pregnancy outcome (*P* = 0.32; % mediated = 16%; **Table 6**).

**Table 6:** Adjusted mediation model of the relationship of cumulative social ACEs on pregnancy outcomes and depression during pregnancy (N = 593)

	Coefficients*				Sobel test		% Mediated†
**Mediator‡**	**a**	**b**	**c**	**c’**	**SE**	***P*-value**	
**Gestational age:**							
Social ACEs	1.89 (8.44)	-0.06 (-3.17)	-0.13 (-1.31)	-0.009 (-0.08)	-0.12 (-2.93)	0.0033	93
**Birth weight:**							
Social ACEs	1.90 (8.44)	-14.66 (-1.99)	-96.69 (-2.69)	-68.86 (-1.79)	-27.82 (-1.92)	0.054	28.8
**Fetal distress:**							
Social ACEs	1.82 (8.41)	-0.02 (-0.99)	-0.25 (-2.23)	-0.21 (-1.73)	-0.04 (-0.98)	0.32	16

ACE – adverse childhood experience

*Model adjusted to the effects of environmental tobacco smoking, high risk pregnancies and intra-familial ACEs.

†% Mediated = (c – c’)/c.

‡Mediator: Depression during pregnancy.

## DISCUSSION

To our knowledge, this is the first study in Tunisia and North Africa that explores the relationship between exposure to social violence and adverse pregnancy outcomes. Our results demonstrate that exposure to social violence during a woman’s first 18 years of life can influence her reproductive health, defined in our analysis as preterm birth, low birth weight, and acute fetal distress. The more social ACEs that women experienced, the greater were their risks of presenting adverse pregnancy outcomes. Our results showed that exposure to social ACEs and experiencing negative pregnancy outcomes, such as low birth weight and fetal distress, were not mediated by the mental health status of the pregnant women. However, premature birth was almost completely mediated by depressive affect during pregnancy.

The relationship of objective pregnancy outcome measures and early life adversity found in our study extends previous findings showing that intra-familial ACEs (child abuse and household dysfunction) are associated with premature delivery and low-birth weight [8,9]. Furthermore, it supports past research [18-20] by confirming the association between maternal history of adversities during childhood and depression during pregnancy. Our analysis adds power to the previous literature on the negative impact of social ACEs on depression. Previous researchers have explored peer violence, which has shown that bullied adolescents were at a greater risk of depressive disorders [24,26]. This includes a dose-response relation between increasing frequency of victimization and violence by peers in adolescence and the risk of developing later depression [27]. Others have also assessed the impact of community violence in childhood on other mental illnesses such as posttraumatic stress disorder symptoms (PTSD) in children and adolescents [28]. Importantly, few have examined community violence in childhood and depression during pregnancy. Those who have, have shown that indirect exposure to community violence predicted PTSD symptoms to the same extent as personal victimization [29]. We extend this body of literature to show that exposure to a wide variety of extreme social violence, including exposure to collective (eg, war) as well as peer and community (eg, protest, civil unrest, or demonstrations) violence, was not only associated with depression during pregnancy, but also predicted birth outcomes.

It is well-established that the mother’s mental health status is tightly linked to pregnancy outcomes and the postnatal period. Some studies [14,39] have demonstrated that women with prenatal depressive disorders are more likely to have pregnancy complications such as low birth weight infants and preterm delivery in comparison with women without such prenatal depressive disorders. Seng et al. [40] confirmed that there is an association between PTSD and important adverse pregnancy outcomes (low birth weight and preterm delivery) and that the effect is much more marked among women with ACEs and subsequent PTSD. Our results suggest that the cumulative burden of social violence was partially mediated by depressive affect, but depressive affect did not mediate low birthweight or fetal distress.

The relationship between ACEs and later perinatal depression is supported by biological mechanisms especially a disruption of the neurobiological response to stress as a dysregulation of the sympathetic nervous system, the serotonin system, and the hypothalamic-pituitary-adrenal axis [41,42].

The novel findings and the strengths of this study are worth noting here. We explored the direct impact of social ACEs on delivery outcomes and demonstrated that both low birth weight and fetal distress are directly related to the severity of social violence exposure. The prospective design and careful analysis and control for alternative explanations are this study’s greatest strengths. We controlled for some important confounds such as personal substance use, exposure to second hand smoke, high-risk pregnancy factors, and past abortions. We are the first to analyze the impact of the full range of social violence adversities on pregnancy outcomes and postpartum period while the majority of researchers have focused only on intra-familial adversities such as physical and sexual abuse. Our findings are valuable regionally in this particular time of transition following the various social and political changes. They call for deployment of strategies to address or prevent the short and long-term potential mental and physical health sequelae in women and children exposed to intra-familial and social violence adversities.

In our study certain limitations have to be pointed out. First, the findings are limited to the sample upon which they were based and may not apply to other countries experiencing social and political unrest. Second, the women consulting primary health care centers generally had a low socioeconomic status, which restricts their generalizability to the rest of social classes. Third, some measures were based on self-reported data, and thus are subject to the various cultural biases inherent in such information. These same biases are inherent in the collection of ACE information, which could affect the validity and reliability these outcomes. Another bias is that some potentially important variables were not measured eg, nutrition, social support, education.

Given that we assured the anonymity of all responses, the social desirability bias may be limited. Past research has shown that self-report adversity is highly correlated with objective social records, thus fairly reliable [43].

## CONCLUSIOND

A healthy pregnancy starts long before conception. We demonstrate here that early life exposure to social violence in mothers is associated with low birth weight and fetal distress at birth. Further, we show that this effect is above and beyond forms of intra-familial adversity, depressive affect, and other risk factors for birth complications. Identifying risk factors for adverse pregnancy outcomes and postnatal depression is of considerable importance given their negative impact on the infant and the mother’s health. Hence, a public health framework based on the collaboration between pediatric, psychiatric, and obstetrical health professionals could be applied to ensure primary prevention of childhood adversities, and secondary prevention of their possible repercussions on mental and physical health of young adolescents before pregnancy. Future projects will include a component focusing on the promotion of resiliency which we hope will help to reduce social violence. Integrating screening and treatment for PTSD and depression into prenatal examination routines would be a reasonable and crucial step in addressing their potential risks on pregnancy outcomes and postpartum depression. More research is needed to better understand the long-term biological mechanisms responsible for the sequelae related to the early life adversities.

## Additional material

Online Supplementary Document
