# Supplementary material for: Impact of social violence and childhood adversities on pregnancy outcomes: a longitudinal study in Tunisia
Source: J Glob Health. 2019 Dec 16;9(2):020435. doi: 10.7189/jogh.09.020435 (PMC6925971; doi:10.7189/jogh.09.020435)
Supplement: Online Supplementary Document [file jogh-09-020435-s001.pdf]

Appendix S1: List of questions related to intra-familial and social ELAs in the Adverse Childhood Experiences-International Questionnaire

ELA Type	No. of questions	Example of questions	Criteria for positive case
Parent/caregiver relationship	2	Parents understand children's problems / worries; know what children do with their free time	Responses of "many times" or "sometimes"
Neglect	4	Insufficient food even when available; parents taking drinks or drugs; refusing to send child to available school; insufficient use of available medical care	Responses of "many times" or "sometimes"
Household dysfunction	1 (2 part)	Part 1 – living with substance abuser, mentally ill, or imprisoned household members; parental separation or death<br><br>Part 2 – witnessing household violence	Response was "yes" to any of Part 1 or "many times" and "sometimes" for the Part 2
Physical abuse	4	Parents push, grab, slap, or throw something at children or hit their children so hard resulting in mark or injury	Responses of "many times" or "sometimes"
Sexual abuse	4	During the first 18 years of life an adult, relative, family friend, or stranger:<br><br>-ever touched or fondled your body in a sexual way<br><br>-had you touch their body in a sexual way<br><br>-attempted to have any type of sexual intercourse with you (oral, anal, or vaginal)<br><br>-effectively had any type of sexual intercourse with you (oral, anal, or vaginal)	Responses of "many times" or "sometimes"

Peer violence	2	Bullied or physical fight	Responses of “many times” or “sometimes”
Community violence	3	Beaten up; stabbed/shot or threatened with a knife/gun in real life	Responses of “many times” or “sometimes”
Collective violence	4	Wars, terrorism, political or ethnic conflicts, genocide, repression, disappearances, torture and organized violent crime such as banditry and gang warfare	Responses of “many times” or “sometimes”
